# SNPS in the exons of toll-like receptors are associated with susceptibility to type 1 diabetes in Chinese population

**DOI:** 10.1186/1687-9856-2015-S1-P24

**Published:** 2015-04-28

**Authors:** Dijing Zhi, Chengjun Sun, Shuixian Shen, Feihong Luo, Carani B Sanjeevi

**Affiliations:** 1 Children's Hospital of Fudan University, Shanghai, China; 2 Karolinska Institutet, Stockholm, Sweden

## Objective

Toll-like receptors (TLRs) recognize a wide range of pathogen-associated molecular patterns (PAMP) and mount the initiation of immune response. Single nucleotide polymorphisms (SNPs) in exons of genes encoding TLRs might be responsible for the generation of an abnormal immune response which could lead to autoimmune diseases. In this study, we investigated the SNPs in TLRs in a Chinese population, and we hypothesized that SNPs in TLRs are associated with type 1 diabetes (T1D), an autoimmune disease caused by destruction of insulin producing pancreatic β-cells, in the studied population.

## Research design and methods

We selected 28 SNPs in exons of TLRs with an aim to identify those that might have a direct correlation with T1D etiology and many have not been included in previous GWAS studies. Genotyping of those SNPs in TLRs was performed in 429 T1D patients and 300 age and gender-matched healthy controls in Chinese Han population. The earlier GWAS studies did not include samples from China.

## Results

Among the SNPs genotyped, the T allele of TLR1-626 was found to be positively associated with T1D (OR=1.98, Pc=0.01). We identified another T1D associated locus in TLR6, the homozygous AA genotype of TLR6-1329 was negatively and heterozygous GA was positively associated with T1D (OR=0.54, Pc=0.02 and OR=1.70, Pc=0.03). We also identified the haplotype T-G-A in TLR1 gene to be positively associated with T1D (OR=2.22, Pc=0.03). Additional haplotypes in TLR-6 also showed significant positive and negative association. In addition, our haplotype analysis and conditional analysis showed that these two SNPs are the primary T1D associated loci among the SNPs tested in our cohort in each TLR gene.

## Conclusion

SNPs and haplotypes in TLR1 and TLR6 gene were associated with T1D in Chinese Han population. Our study, for the first time, indicates that TLR1 and TLR6 gene might play important roles in the etiology of T1D.

